# Hardware removal rates after surgical treatment of proximal femur fractures: Nationwide trends in Finland in 1997–2016

**DOI:** 10.1007/s00402-020-03356-z

**Published:** 2020-01-21

**Authors:** Ville T. Ponkilainen, Tuomas T. Huttunen, Pekka Kannus, Ville M. Mattila

**Affiliations:** 1 Department of Orthopaedics and Traumatology, Tampere University Hospital, Kuntokatu 2, 33520 Tampere, Finland; 2 Faculty of Medicine and Health Technology, Tampere University, Tampere, Finland; 3 Department of Emergency, Anesthesia and Pain Medicine, Tampere University Hospital, Tampere, Finland; 4 The Division of Orthopedics and Biotechnology, Department of Clinical Science, Intervention and Technology (CLINTEC), Karolinska Institutet, Stockholm, Sweden; 5 COXA Hospital for Joint Replacement, Tampere, Finland

**Keywords:** Fixation, Material, Removal, Secondary, Endoprosthesis

## Abstract

**Background:**

Various internal fixation methods have been used to treat proximal femur fractures and occasionally the fixation material is removed. However, nationwide trends of hardware removals are not known. Thus, this study investigated the hardware removal rates after proximal femur fractures in Finland during 1997**–**2016.

**Materials and methods:**

Finnish adults aged 18 years or older in 1997**–**2016 formed the basic study population. From the National Hospital Discharge Register patients with trochanteric femur fracture treated with an intramedullary nail (IMN) or dynamic hip screw (DHS), and patients with femoral neck fracture treated with screw fixation, were included. Hardware removal and secondary prosthesis rates were assessed.

**Results:**

Altogether 41,253 patients underwent proximal femoral fracture fixation surgery in Finland in 1997**–**2016. Of these, 16,152 were DHS surgery and 15,724 IMN surgery and 8491 underwent screw operation of femoral neck fracture. The total removal rates of DHS and IMN were 5.5% and 5.4%. The total removal rate of screw fixations of the femoral neck was higher, 18.5%. The total removal rates during the first 3 years after the IMN more than halved in 1997**–**2013, from 7.6% to 3.7%, whereas the removal rate of the DHS or screw fixation of femoral neck fractures did not show consistent trend. The rate of secondary prosthesis operations following DHS and IMN was low (1.8% for both). This was in clear contrast to the prosthesis rate following screw fixations of the femoral neck (7.2%).

**Conclusions:**

IMN operations largely replaced DHS operations in trochanteric fractures of the proximal femur in Finland in 1997**–**2016. The removal and secondary prosthesis rates of the DHS and IMN were clearly lower than the corresponding rates after screw fixations of the femoral neck fracture.

**Level of evidence:**

III, Epidemiologic study.

## Introduction

Fractures of the proximal femur are one the most common age-related fractures in older adults and in entire populations they represent the third most common fracture type after distal radius and metacarpal fractures [12]. Proximal femur fractures are very costly [18, 36, 43] with high post-fracture morbidity and mortality [10, 26]. Women have an increased risk for these fractures and the fracture incidence increases with age, propensity to fall and reduced bone density [22, 23, 26, 29, 42].

The incidence of proximal femur fractures increased rapidly till the end of 1990s, although there was notable variation between different countries [12, 23, 35, 40]. In the new millenium, the incidence has stabilized or even decreased [13, 16, 22, 25, 30, 45]. The proportion of trochanter fractures from all proximal femur fractures has increased and this is especially true among older age groups [16, 33, 38, 39, 45].

Various fixation methods have been used in the treatment of trochanteric fractures [4, 7, 14, 46, 48]. Dynamic hip screw (DHS) has been the most preferable method for years [46], although intramedullary nail (IMN) has been adopted to routine use of these fractures as well [1, 8]. Recent meta-analyses have favored the use of IMN, since post-operative mobilization is faster with IMN than DHS [3, 24, 41].

The treatment of femoral neck fractures has remained controversial for decades [11]. Osteosynthesis with internal screw or pin fixation has been used in non-displaced or minimally displaced fractures while primary arthroplasty has been suggested for displaced fractures [37]. Recent meta-analyses have shown that arthroplasty is slightly superior when compared to internal screw fixation of these fractures [2, 32, 44].

Removal of fixation material has accounted for 5**–**15% of all orthopaedic surgeries and up to 30% of all elective orthopaedic surgeries [6, 47]. In Finland, this number has been 6.3% of all orthopaedic operations representing an incidence of 90 per 100,000 person-years [6]. Nevertheless, the removal of the fracture fixation material should not be a routine procedure [9]. These procedures cause unwanted health care costs and sick leaves, and they cause a risk for post-operative infections, wound healing problems, refractures, nerve damage, and long-term pain [9, 15, 19]. In general, the literature investigating fixation material removals is scarce and there are no evidence-based guidelines for fixation material removals and the removal policy varies widely among professionals [20].

The aim of this study was to investigate the fixation material removal rates after surgery for proximal femur fractures in Finland between 1997 and 2016.

## Methods

Patient data were obtained from the Finnish National Hospital Discharge Register (FHDR) between 1997 and 2016. All adult patients aged 18 years or older were included into study. Patient characteristics, such as age, sex, primary and secondary diagnosis, and operations performed during the hospital stay, were obtained from the database. The coverage and accuracy of the database have been shown to be excellent [27, 34, 49]. The FHDR does not include co-morbidities and other risk factors for fractures; thus this study focused on incidence rates.

Patients were selected using the diagnoses which have been coded with the International Classification of Diseases, Tenth Revision (ICD-10). The diagnoses were then combined with the NOMESCO (Nordic Medico-Statistical Committee) classification procedure codes. Only patients with fractures treated with osteosynthesis were included. From Finnish NHDR, we included: Code S72.0 (Fracture of neck of femur), which was combined with NFJ50 (Internal fixation of fracture of neck of femur with nail or screw), and code S72.1 (Pertrochanteric fracture), which was combined with NFJ54 (Internal fixation of fracture of upper femur with intramedullary nail) or NFJ52 (Internal fixation of fracture of upper femur with screws and sideplate). Among these patients, all fixation material removals which were performed after the initial surgery were identified using the NOMESCO code NFU20 (Removal of internal fixation device from femur).

The delay between the primary procedure and removal of fixation material was calculated using the admission date between the two hospitalizations. If the same patient underwent multiple hospitalizations due to the same operation, only the first one was included, as we were unable to tell whether the second admission was due to a new fracture or a complication of the first proximal femur fracture. Patients who underwent multiple different primary hip operations were excluded, since we are unable to know which fixation material was removed. If a patient underwent removal and prosthesis operations during the same hospital stay, this hospital stay was considered as secondary prosthesis operation.

### Statistical analysis

The incidence rates of the operations were based on the annual mid-populations, which were obtained from the Official Statistics of Finland. The incidences rates (per 100,000) were based on the entire adult population of Finland (persons 18 years of age or older). R (version 3.6.1) statistics software were used for statistical analysis.

## Results

**Fig. 1:**
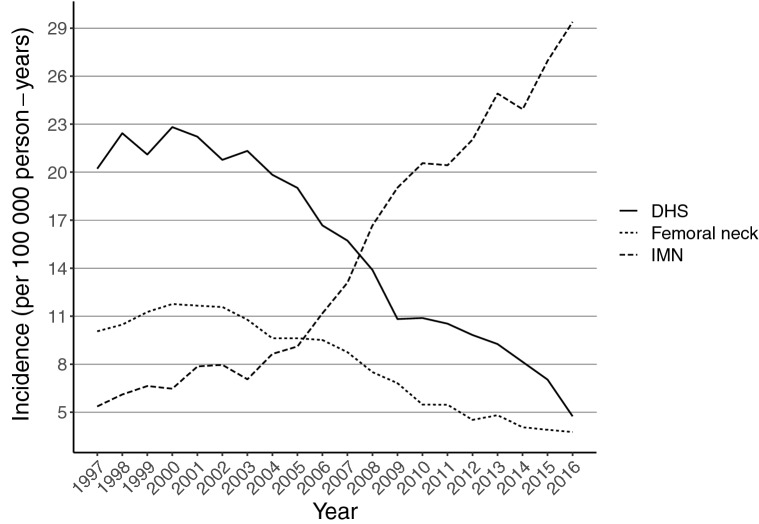
Incidence of screw fixation for femoral neck fracture, and dynamic hip screw (DHS) and intramedullary nail (IMN) fixation for trochanteric fracture of the proximal femur

Altogether 41,253 patients underwent proximal femoral fracture fixation operations in Finland between 1997 and 2016. Of these, 16,152 were DHS surgery and 15,724 IMN surgery due to trochanteric fracture of the femur, and 8491 underwent screw operation due to femoral neck fracture. During the study period DHS operations declined from 20 per 100,000 person-years in 1997 to 5 per 100,000 person-years in 2016 while IMN operations increased remarkably, from 5 per 100,000 person-years in 1997 to 29 per 100,000 person-years in 2016 (Fig. 1). Screw fixations of femoral neck fractures declined from 10 per 100,000 person-years in 1997 to 4 per 100,000 person-years in 2016.

**Fig. 2:**
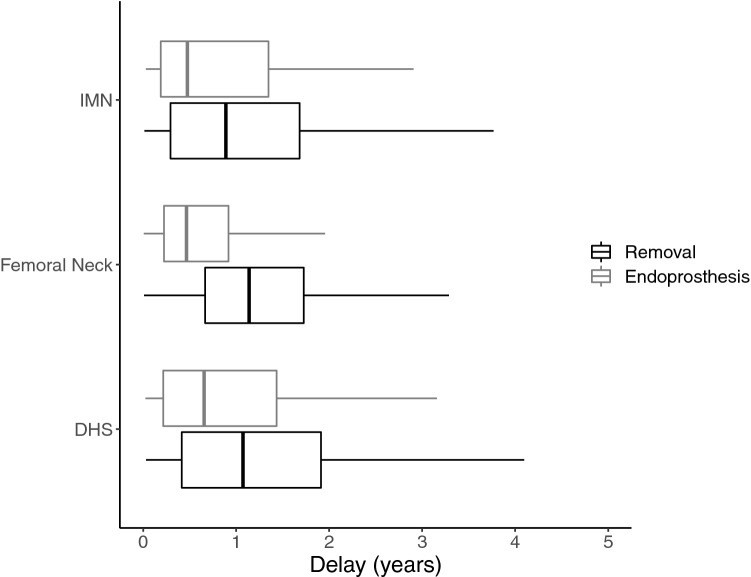
Boxplot of time intervals between the primary and the two secondary operations of the proximal femur fracture. The category “endoprosthesis” consisted of hardware removal and prosthesis surgery during the same hospital stay

The total removal rates of DHS and IMN were rather similar (5.5% and 5.4%). However, the total removal rate after screw fixations of femoral neck fracture was higher (18.5%) (Table 1). The proportion of removals (without prosthesis) of DHS and IMN were relatively similar (3.7% and 3.6%). The similar removal rate after screw fixations of femoral neck fracture was higher (11.3%) (Table 1). Similarly, the rates of removals with secondary prosthesis operations following DHS and IMN were the same (1.8%). This was in clear contrast to the rate following screw fixations of femoral neck fractures (7.2%) (Table 1). The secondary prosthesis operations (which consisted of hardware removal and prosthesis surgery during the same hospital stay) were performed after a shorter time period than the fixation material removals (Fig. 2).

**Table 1:** Total operations, secondary operations and removal delays after proximal femur fracture fixation operations

	*n*	%	Delay of the removal (years)			
			< 1	1–2	2–5	5 +
DHS	16,152					
Secondary operations	895	5.5	441	218	168	68
Removals	598	3.7	270	160	123	45
Prosthesis	297	1.8	171	58	45	23
IMN	15,724					
Secondary operations	844	5.4	461	192	143	48
Removals	564	3.6	287	149	100	28
Prosthesis	280	1.8	174	43	43	20
Screw fixation	8491					
Secondary operations	1574	18.5	814	420	245	95
Removals	959	11.3	384	344	177	54
Prosthesis	615	7.2	430	76	68	41

*DHS* dynamic hip screw for trochanteric fracture, *IMN* intramedullary nail for trochanteric fracture, *Screw fixation* screw fixation for femoral neck fracture, *Removals* removal of fixation materials, *Prosthesis* removal of fixation materials and endoprosthesis

**Fig. 3:**
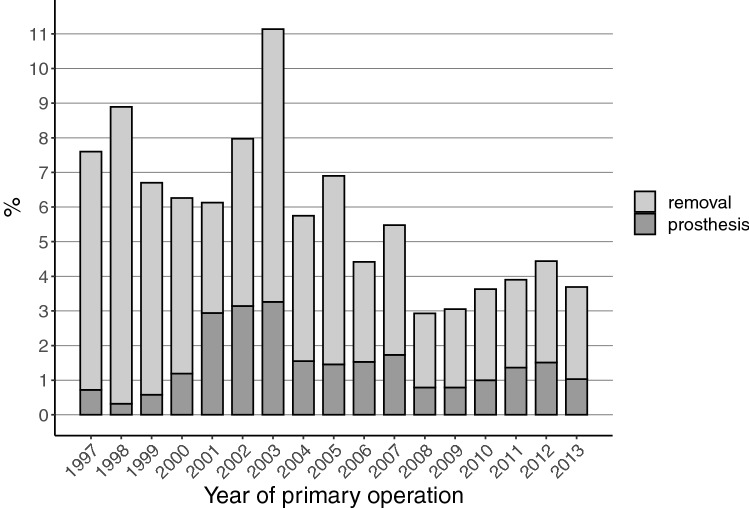
Total hardware removal and prosthesis rates during the first three years after intramedullary nail fixation of the trochanteric fracture of the proximal femur

The total removal rates of the fixation material during the first three years after the IMN operation halved during the follow-up, from 7.6% (1997) to 3.7% (2013) (Fig. 3). The prosthesis implantation rates after the IMN surgery did not show any consistent trend during the study period.

**Fig. 4:**
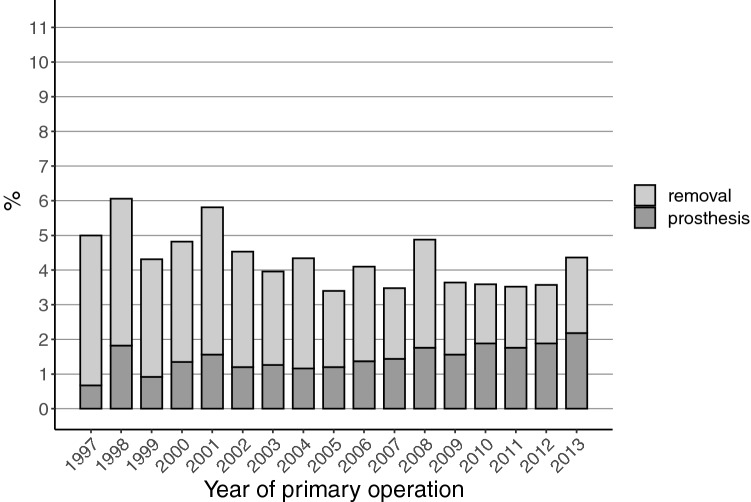
Total hardware removal and prosthesis rates during the first three years after dynamic hip screw fixation of the trochanteric fracture of the proximal femur

The total removal rates of the fixation material during the first 3 years after the DHS operation decreased slightly (from 5.0% in 1997 to 4.4% in 2013) during the study period (Fig. 4). The prosthesis implantation rates after the DHS surgery increased during the study period, from 0.7% in 1997 to 2.2% in 2013.

**Fig. 5:**
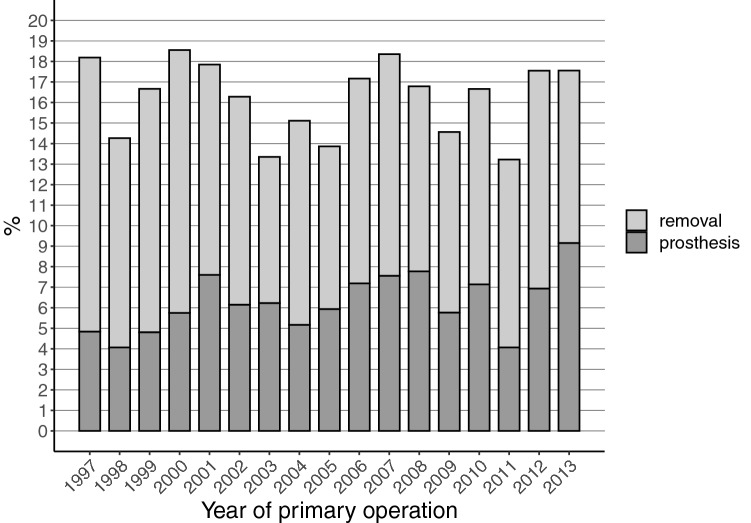
Total hardware removal and prosthesis rates during the first three years after screw fixation of the fracture of the femoral neck

The total removal rates of the fixation material during the first 3 years after the screw fixation of femoral neck fractures did not show consistent trend changes during the study period (18.2% in 1997 vs. 17.6% in 2013) (Fig. 5). The prosthesis implantation rates after this screw fixation increased during the study period, from 4.8% in 1997 to 9.2% in 2016.

## Discussion

This study showed that IMN operations largely replaced DHS operations as the treatment for trochanteric fractures of the proximal femur in Finland during the years 1997 and 2016 while screw fixation of femoral neck fractures decreased during these years. The total removal rates of DHS and IMN were rather similar (5.5% and 5.4%), while the total removal rate of screw fixations of femoral neck fracture was clearly higher (18.5%). The rate of secondary prosthesis operations following DHS and IMN was low (1.8% for both). This was in clear contrast to the prosthesis rate following screw fixations of the femoral neck fractures (7.2%).

Historically, various fixation methods have been suggested for treatment of proximal femur fractures [4, 7, 14, 46, 48]. However, problems with failure of the fixation, nonunion, and high mortality encouraged to develop more efficient techniques [7, 14]. Dynamic hip screw (DHS) was introduced for trochanteric fractures to solve the problems regarding nonunion and the results were favorable [46]. When intramedullary nails (IMN) for trochanteric fractures were first introduced, it was noted that the risk for secondary femoral shaft fractures were higher than when using DHS, and therefore it was not at first adapted to routine use [1, 8]. However, recent meta-analyses have shown that the problem with femoral shaft fractures has been resolved and the use of IMN nails does not have a higher risk for complications than DHS [3, 24]. Furthermore, a recent randomized prospective study showed that patients treated with IMN were able to return their pre-surgery walking ability faster than patients treated with DHS [41].

The controversy between internal fixation and primary arthroplasty for optimal treatment of displaced femoral neck fractures has existed for decades [11]. Recent meta-analyses have shown that the primary arthroplasty is slightly superior to internal fixation in terms of revision surgery, infection rate, pain, blood loss and operation time [2, 32, 44]. The results of our study support the findings from these meta-analyses: the incidence of screw fixations of femoral neck fractures declined from 10 per 100,000 person-years in 1997 to 4 per 100,000 person-years in 2016. In other words, it seems that primary arthroplasty is displacing internal fixation as the first-line treatment of displaced femoral neck fractures.

Originally, various adverse effects, such as deep late infection, metal corrosion, pseudotumors and even neoplasia, were proposed to be the result from fixation material, and this doubt led towards routine removals [5, 17, 21, 28, 50]. On the other hand, it has been noted that these problems are not commonly related to modern fixation materials and the routine removals of the fixation material have been criticized [9]. As lack of evidence in the literature has remained, the removal rates have varied between professionals and areas [9, 20].

Lovald et al. published a study investigating the fracture fixation material removals in the USA during 2007 [31]. In their study, the removal rate was calculated from data of 1 year, and they reported a removal rate of 15.8%. The most common reasons for the fixation material removals were mechanical complications (18.7%), osteoarthritis (14.3%), nonunion (13.9%), refracture (10.9%), and other complications (10.1%) [31]. Interestingly, they also reported that the likelihood of fixation material removal was affected by age, sex and insurance status [31].

In our study, the total removal rate for IMN and DHS of the intertrochanteric fracture was 5.5%, and for screw fixation of the femoral neck fractures 18.5%. The removal rates of all internal fixation methods decreased or stayed relatively stable throughout the years. Thus, our removal rates for IMN and DHS were remarkably lower than previously reported (16%) [31]. One of the reasons for our lower figures could be that our publicly funded health care system has an intrinsic aim for cost-savings and cost-effectiveness thus preferring non-removals.

A remarkable change in our study occurred in IMN removals, which decreased about 50% between 1997 and 2013. This change seems to be in line with the literature, as no new studies supporting the removals have been published. We also noted a slightly increasing trend that in fractures of the femoral neck the screw fixation material is replaced with an endoprosthesis (Fig. 5). This, however, is a moribund phenomenon since incidence of screw fixation for femoral neck fractures is drastically declining (Fig. 1).

The strength of our study was the nationwide National Hospital Discharge Register which included all fixation operations for proximal femur fractures in Finland between 1997 and 2016. Every patient had a personal ID number and we were able to follow these numbers through the study period. In this way, we could also calculate precise removal rates of the fixation material for the entire surgically treated fracture population. The limitation of this study was that indications for the removals were not registered, and therefore, the exact reasons for the observed removal trends remained unknown.

As a conclusion, IMN operations increased in Finland in 1997–2016 and largely replaced DHS operations as the treatment of choice for trochanteric fractures of the proximal femur. At the same time, screw fixation of femoral neck fractures decreased. The total removal and secondary prosthesis rates of DHS and IMN were similar (total removal rates 5.5.% and 5.4%, prosthesis rate 1.8% for both), while the total removal and secondary prosthesis rates of screw fixations of femoral neck fracture were clearly higher (removal rate 18.5%, prosthesis rate 7.2%). The total removal rates of the fixation material during the first three years after the IMN operation halved during the follow-up, while the removal rates of DHS and screw fixation for femoral neck fractures did not show any consistent time trend.
